# Commencement of the College Course

**Published:** 1872-09

**Authors:** 


COMMENCEMENT OF THE COLLEGE-COURSE.—The Preliminary Term in the Buffalo Medical College will commence on Wednesday, Oct. 9th. This term is to a large extent devoted to Practical Anatomy; Clinical lectures will however, be given in the Hospitals by Professors Rochester and Miner on Wednesday and Saturday of each week. The regular Term of Lectures will commence Nov. 6th. From present indications an unusually large class may be expected.

